# Psychological Interventions for the Management of Glycemic and Psychological Outcomes of Type 2 Diabetes Mellitus in China: A Systematic Review and Meta-Analyses of Randomized Controlled Trials

**DOI:** 10.3389/fpubh.2015.00252

**Published:** 2015-11-16

**Authors:** Anna Chapman, Shuo Liu, Stephanie Merkouris, Joanne C. Enticott, Hui Yang, Colette J. Browning, Shane A. Thomas

**Affiliations:** ^1^School of Primary Health Care, Monash University, Notting Hill, VIC, Australia; ^2^Royal District Nursing Service Institute, Melbourne, VIC, Australia; ^3^Beijing Office for Cancer Prevention and Control, Peking University Cancer Hospital and Institute, Beijing, China; ^4^School of Psychology, Deakin University, Melbourne, VIC, Australia; ^5^School of Clinical Sciences, Monash University, Melbourne, VIC, Australia

**Keywords:** type 2 diabetes mellitus, psychological intervention, China, therapy, systematic review, meta-analysis, motivational interviewing, cognitive behavioral therapy

## Abstract

**Introduction:**

China has the largest number of type 2 diabetes mellitus (T2DM) cases globally, and T2DM management has become a critical public health issue in China. Individuals with T2DM have an increased risk of developing mental health disorders, psychological disturbances, and functional problems associated with living with their condition. Previous systematic reviews have demonstrated that, generally, psychological interventions are effective in the management of T2DM-related outcomes; however, these reviews have predominantly included studies conducted within English-speaking countries and have not determined the efficacy of the varying types of psychological interventions. As such, this paper aims to synthesize evidence and quantify the efficacy of psychological therapies for the management of glycemic and psychological outcomes of T2DM in China, relative to control conditions.

**Methods:**

A systematic search (MEDLINE, PsycINFO, CINAHL, Cochrane Central Register of Controlled Trials, China National Knowledge Infrastructure, and Wangfang Data) for all years to December 2014 identified all available literature. Eligibility criteria included: peer-reviewed journal articles, randomized controlled trials (RCTs) assessing the efficacy of a psychological therapy for the management of T2DM, adult participants (≥18 years) diagnosed with T2DM or non-insulin-dependent diabetes mellitus, and Chinese speaking participants only (in mainland China). Outcome measures were glycated hemoglobin, blood glucose concentration, depression, anxiety, and quality of life. Effect sizes were pooled using a random effects model. Negative effect sizes corresponded to positive outcomes favoring the intervention.

**Results:**

Forty-five RCTs were eligible for the meta-analyses. Cognitive behavioral therapy (CBT) and motivational interviewing (MI) were more effective than the control condition in the reduction of glycated hemoglobin [CBT: −0.97 (95% CI −1.37 to −0.57); MI: −0.71 (95% CI −1.00 to −0.43)]. CBT and client-centered therapy (CCT) were also associated with reductions in depression and blood glucose concentration, and CBT was associated with reductions in anxiety.

**Conclusion:**

Psychological interventions, namely, CBT, MI, and CCT are effective in improving certain T2DM-related outcomes in China. Considerable levels of heterogeneity and unclear risk of bias associated with most included RCTs suggest caution when interpreting results. In China, where the burden of T2DM is increasing significantly, psychological interventions may provide promising approaches to assist in the management of T2DM to delay the progression of T2DM related outcomes.

## Introduction

Type 2 diabetes mellitus (T2DM) is a complex metabolic condition that requires effective long-term management in order for patients to achieve optimal glycemic control and to prevent chronic complications (1). As lifestyle, behavioral, and psychological changes are fundamental to the management of T2DM, it is essential for health care teams and patients to work collaboratively to ensure patients adhere to clinical recommendations and the T2DM self-care regimen (2). Internationally, evidence-based guidelines recognize the importance of a structured and systematic approach to the management of T2DM in the primary care setting that incorporates psychological care within clinical recommendations (3–5). It is also widely recognized that compared to the general population, individuals with T2DM have a higher prevalence of clinical and sub-clinical levels of depression and anxiety (6, 7), with the potential consequences being reductions in glycemic control, quality of life, and treatment adherence (7, 8).

Type 2 diabetes mellitus in China has reached epidemic proportions and has largely been attributed to the rapid urbanization China has experienced over the past two decades. This urbanization has triggered significant improvements in living conditions and socioeconomic status for Chinese residents and has resulted in reductions in physical activity and increases in high energy, high fat diets, body mass index, and central upper-body adiposity (9). With the largest global population of diabetes mellitus (DM), the International Diabetes Federation has advised that China will face significant challenges in its response to DM (10). Recent data suggests that 96.3 million adults in China have DM, and this figure is expected to rise to 142.7 million by 2035 if no action is taken (10).

Typically, chronic illness management approaches in China are not patient centered, nor do they include a central role for the patient in the self-management of their condition (11). Furthermore, patients are often dissatisfied with the medical services they receive while doctors focus on providing medications to manage chronic diseases rather than the facilitation of behavior change to moderate or control these conditions (12, 13). Doctors and nurses in China are not specifically trained in psychology, and their skills are often limited in the counseling and behavior change techniques that have been recognized as having the potential to assist patients in adhering to the lifestyle changes necessary for effective management of T2DM (14).

Previous systematic reviews and meta-analyses (15–17) have demonstrated that, generally, psychological interventions can be effective in the management of T2DM, through the examination of key outcomes including glycated hemoglobin and psychological status. However, these reviews were limited by assessing the effectiveness of psychological interventions as a whole, with no distinction between the varying therapeutic types. Furthermore, the effectiveness of psychological interventions in the previous reviews has largely been based on research conducted within English-speaking countries. To date, no reviews have focused on the effectiveness of psychological interventions of T2DM in China, and previous reviews have only utilized international databases [that have limited indexation of Chinese-language journals (18)] in their search strategies. Given that the majority of research conducted in China is published in Chinese-language journals (19), there is considerable risk that many pertinent studies may have been overlooked.

With such large and increasing numbers of people with T2DM in China, and the ensuing research that is being conducted in China to address this issue, it is necessary that a review of psychological interventions for the management of T2DM focusing on China be performed. By conducting a comprehensive assessment of relevant T2DM management interventions, future and effective interventions can be tailored to meet the needs of a growing T2DM population in China. As such, this paper presents the first systematic review and meta-analyses of psychological interventions for the management of glycemic and psychological outcomes of T2DM in China that uses both international and Chinese-language databases, in order to quantify the efficacy of interventions relative to control conditions.

## Methods

This review is reported according to the preferred reporting items for systematic reviews and meta-analyses (PRISMA) guidelines (20).

### Data Sources and Search Strategy

A systematic search identified all available studies assessing the efficacy of psychological interventions for the management of T2DM in China. Electronic databases were searched for both internationally published literature (MEDLINE, PsycINFO, CINAHL, and Cochrane Central Register of Controlled Trials) and Chinese-language literature (China National Knowledge Infrastructure and Wangfang data – the two most comprehensive Chinese academic databases). A combination of keywords, wildcards, and appropriate truncation for each database relating to DM (“Diabetes Mellitus” “
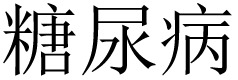
”), psychological interventions (“Psych$” “
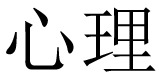
” or “Therap$” “
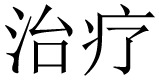
” or “Counsel$” “
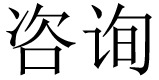
” or “Self-manag$” “
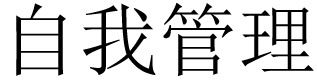
”), and China (“China” or “Chinese” “
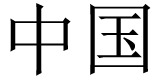
”) was used. The following Chinese journals were also searched manually: *Chinese Mental Health Journal* (1994 onward) and *Shanghai Archives of Psychiatry* (1994 onward). The reference lists of included studies and relevant reviews were searched manually. The search was restricted to peer-reviewed journal articles for all years up to December 2014. First, four authors (AC and SM for international databases and SL and HY for Chinese databases) independently assessed titles and abstracts of studies for inclusion. Second, both groups of authors compared selected studies for inclusion. When discrepancies occurred, consensus was reached through discussion with all four assessing authors (AC, SM, SL, and HY).

### Inclusion Criteria

Eligibility criteria included randomized controlled trials (RCTs) of a psychological intervention, adult participants (≥18 years) with a diagnosis of T2DM or non-insulin-dependent DM, and Chinese-speaking participants only (in mainland China). Studies that did not specifically identify the type of DM or combined types of DM, not separated in analysis, were excluded from the review.

The most commonly used psychotherapeutic models for the management of T2DM were used to categorize psychological interventions. These were cognitive behavioral therapy (CBT), motivational interviewing (MI), client-centered therapy (CCT), brief psychodynamic psychotherapy (BPP), and interpersonal psychotherapy (IPT) (see Box 1 for definitions). Studies that clearly labeled the therapeutic model used in their intervention were accepted at face value. Where interventions were not clearly labeled, these studies were included and categorized if they used one or more psychological techniques that could be classified as a component of a specific psychotherapy (e.g., relaxation training classified as a component of CBT). This process was performed independently by two authors (AC and SM), and when discrepancies occurred, consensus was reached by consulting a third author (SL). Studies that did not clearly describe the psychological component of the intervention were excluded. Appropriate control groups were defined as non-psychological and included usual care, waitlist, and education. According to the Chinese Guideline for Diabetes Prevention and Management (21), usual care refers to quarterly doctor consultations and completion of an annual physical examination. Control groups that experienced a less intensive psychological therapy were excluded.

Box 1 **Definition of psychological interventions.**

*Cognitive Behavioral Therapy (CBT)* (42): CBT is a generic term referring to therapies that incorporate both behavioral interventions (direct attempts to reduce dysfunctional emotions and behavior by altering behavior) and cognitive interventions (direct attempts to reduce dysfunctional emotions and behavior by altering individual appraisals and thinking patterns). In the present study, techniques, such as cognitive restructuring, emotional adjustment/rational emotive therapy, relaxation, biofeedback relaxation training, music relaxation therapy, goal setting, stress management, and problem solving, were classified as components of CBT.

*Motivational Interviewing (MI)* (43): A non-manualized, individually tailored therapy defined as “a collaborative, goal-oriented style of communication with particular attention to the language of change. It is designed to strengthen personal motivation for and commitment to a specific goal by eliciting and exploring the person’s own reasons for change within an atmosphere of acceptance and compassion.”

*Client-Centered Therapy (CCT)* (44): A non-directive and supportive counseling approach, the goals of CCT are increased self-esteem and openness to experience. Additionally, CCT aims to increase self-understanding; reduce defensiveness, guilt, and insecurity; establish more positive and comfortable relationships with others; and increase capacity to experience and express feelings.

*Brief Psychodynamic Psychotherapy (BPP)* (45): BPP applies the principles of psychoanalytic theory to the treatment of selected disorders, typically within a time frame of 10–25 sessions, from which a manual can be followed. A time limit is determined at the outset of therapy that sets in motion psychological expectancies regarding when change is likely to occur.

*Interpersonal Psychotherapy (IPT)* (46): IPT is a brief, time-limited, and highly structured manual-based psychotherapy that aims to alleviate patients’ suffering and improve their interpersonal functioning. IPT focuses specifically on interpersonal relationships as a means of bringing about change, with the goal of helping patients improve their interpersonal relationships or change their expectations about them.

The primary outcome measure was glycated hemoglobin. Additional outcomes included fasting blood glucose concentration, depression, anxiety, and overall quality of life (QOL) as measured by validated tools. With regard to depression and anxiety, both diagnostic and symptom severity tools were considered appropriate for inclusion. Studies were excluded if they did not report any outcome measures within scope. For multiple reports of a primary study, we maximized the yield of information by collating all data. The publication reporting the longest follow-up was assigned priority.

### Data Extraction

Data from included studies were extracted and translated into English using a standardized template by two authors (HY and SL) who are fluent in English and Mandarin. This included study and sample characteristics (i.e., clinical subgroups and sample size), design features (i.e., therapy type, delivery mode, duration, and regimen of treatment conditions), and study results (i.e., means and SD). Only data reported in the original papers were used for extraction, and no attempts were made to contact authors for additional information.

### Risk of Bias

The Cochrane Collaboration’s tool for assessing risk of bias was utilized to examine the risk of bias in each included study (22). This tool examined randomization procedure, allocation concealment, blinding of outcome assessors, incomplete outcome data, and selective outcome reporting. Two independent authors (AC and SM) assessed risk of bias, and where there was disagreement, the third author served as an arbiter (SL).

### Data Synthesis and Analysis

Separate meta-analyses were conducted for each psychological intervention and outcome of interest, where a minimum of three studies per outcome was considered adequate. The results of all other outcomes were discussed narratively.

Data from included studies were analyzed using RevMan 5.3 software (23). The outcome data closest to 3 months follow-up was used to maximize consistency across the studies. Where possible, the data for variables were converted to the same unit, namely, blood glucose concentration from milligram per liter to millimole per liter.

The standardized mean difference (SMD) was used for continuous outcome variables, to account for the use of different scales or methods of measuring the outcomes of interest (e.g., different scales to assess depression).

Effect sizes were pooled using a random effects model as heterogeneity between the studies was expected. A random-effects model assumes that the variability between the studies is true and provides a more conservative estimate of effect by integrating these differences when calculating the pooled estimate of effect, typically resulting in wider confidence intervals (24). Negative effect sizes corresponded to positive outcomes for the intervention compared with the control (i.e., reductions in depressive symptoms).

The estimated combined treatment effect in absolute units was obtained for the primary outcome measure by multiplying the SMD with the overall pooled estimate of SD.

Heterogeneity was examined using the χ^2^ test for heterogeneity and associated significance value (*P*-value). Due to the limitations with this test when there are low number of studies and/or small sample sizes, *P* > 0.10 was used to indicate statistically significant heterogeneity (24, 25). The *I*^2^ statistic was also examined to determine the degree of heterogeneity, with the following classifications used: not important (0–40%), moderate heterogeneity (30–60%), substantial heterogeneity (50–90%), and considerable heterogeneity (75–100%) (24).

Where there were sufficient numbers of studies (*k* = 10), subgroup analyses were conducted to explore the potential factors contributing to heterogeneity. These were selected *a priori* and consisted of: clinical subgroup (T2DM only vs. T2DM and comorbidity), treatment modality (individual vs. individual and group), classification of treatment type (author defined vs. reviewer defined), and timing of glycated hemoglobin outcome assessment (≤2 months vs. >2 months) (for HbA1c only).

To examine the presence and extent of publication bias and small study effects, funnel plots and the Egger’s test (26) were performed in Stata (Version 12) when there were sufficient numbers of studies in the meta-analysis (*k* = 10). A *P*-value of ≤0.05 was considered representative of statistically significant publication bias (26).

## Results

### Search Results

**Figure 1:**
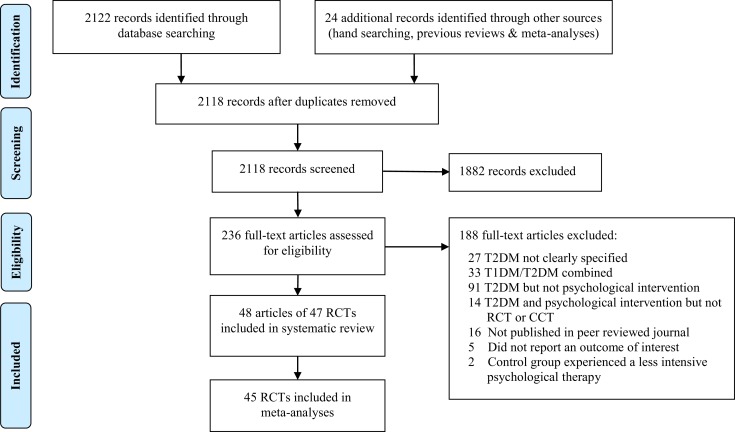
**PRISMA flow diagram**.

Once duplicates were removed, the search yielded 2,118 citations from which 236 full text articles were examined (Figure 1). Of these, 48 articles from 47 RCTs were included in the systematic review and 45 RCTs in the meta-analyses.

Characteristics of included studies are displayed in Table 1. All but one article (27) (a secondary reference) was published after 2000. Both men and women were included in the samples of all RCTs, and sample size ranged from 59 to 598 (119 ± 101). The mean age of participants was 52.8 ± 11.8 years. General T2DM patients were the most commonly specified clinical group (*k* = 35), followed by patients with comorbid depression and/or anxiety (*k* = 10). Mean duration of T2DM was 5.2 ± 3.8 years, and mean duration of follow-up (which typically coincided with intervention duration) was 3.2 months. Most RCTs used either CBT (*k* = 12) or its associated techniques (*k* = 25), with the most commonly used being relaxation training. Five RCTs utilized MI, and five utilized CCT. There were no RCTs that utilized BPP or IPT. No RCTs reported using manualized therapies. Usual care was the most frequently used control condition (*k* = 43). The majority of RCTs were conducted within a hospital setting (*k* = 43).

**Table 1:** **Characteristics of studies included in the systematic review of RCTs examining the effects of psychological interventions for patients with type 2 diabetes mellitus**.

Reference	Number of participants recruited/at follow-up	Mean age ± SD (or range) (years)	Clinical subgroup	Mean duration of diabetes ± SD (or range) (years)	Setting	Model and duration of therapy in intervention group	Regimen in intervention group and specialty of therapist	Model and duration of therapy in control group	Regimen in control group and specialty of therapist	Follow-up (months from baseline)	Outcome measures of relevance
(47)	60/60	72.9 ± 9.6	Depression	8.6 ± 3.0	Hospital (I)	Individual CBT^a^ for 2 months	CBT sessions consisting of relaxation training, music relaxation therapy, emotional adjustment, and psychological support by nurse (number of sessions and manualization NS).	UC for 2 months	Usual nutrition and medication for DM and depression by doctor and nurse (regimen NS)	2	FBG and HAM-D
(30)^b^	100/95	NS	General	NS	Community health	Individual MI for 12 months	31 MI sessions (therapist and manualization NS)	UC for 12 months	UC (regimen and therapist NS)	12	WHOQoL-BREF
(48)	105/105	NS	General	NS	Hospital (I and O)	Individual CBT^a^ for 2 months	8 CBT sessions consisting of morita therapy, emotional adjustment, and psychological support (therapist and manualization NS)	UC for 2 months	Usual medication (regimen and therapist NS)	2	QOL-I
(49)	80/80	47.7 ± 10.8	Depression and anxiety	NS	Hospital (I)	Individual CBT^a^ and family therapy for 2 weeks	CBT sessions consisting of emotional adjustment, music relaxation therapy, psychological support, and family therapy by doctor and nurse (number of sessions and manualization NS)	UC for 2 weeks	UC consisting of nutrition, medication, education, and supportive psychological nursing by nurse (regimen NS)	0.5	SAS, SDS
(50)	72/72	66.0 ± 17.4	General	6.8 ± 14.9	Hospital (I)	Individual CBT^a^ for 2 months	20 CBT sessions consisting of rational emotive therapy and relaxation training (therapist and manualization NS)	UC for 2 months	UC and education by doctor and nurse (regimen NS)	2	SAS, SDS
(28)	60/60	41.5 ± 8.1	General	2.3 ± 0.2	NS	Individual MI and exercise therapy for 6 months	6 MI sessions and 72 sessions of moderate intensity exercise training all by nurse (manualization NS)	Exercise therapy and education for 6 months	72 sessions of moderate intensity exercise training (therapist NS), 6 education sessions consisting of the benefits of exercise and exercise-related knowledge (therapist NS)	6	HbA1c, FBG, and DQoL
(51)	60/60	53.0 ± 10.6	Depression and anxiety	6.4 ± 3.1	Hospital (I and O)	Individual CBT and group education for 1 month	8 CBT sessions consisting of behavior modification, planning, stress management, and psychological support (therapist NS), group education (number of sessions, therapist, and manualization NS)	UC for 1 month	Usual nutrition, exercise, and medication (regimen and therapist NS)	1	HbA1c, FBG, SAS, and SDS
(52)	132/132	49.6 ± 12.0	General	6.1 ± 3.1	Hospital (I)	Individual CBT for 1 month	8 CBT sessions consisting of behavior modification, relaxation training, music relaxation therapy, psychological support, and family therapy by psychologist (manualization NS)	UC for 1 month	Usual medication/insulin treatment (regimen and therapist NS)	1	HbA1c, FBG, SAS, and SDS
(53)	62/62	58.6 ± 7.5	Depression	6.1 ± 2.6	Community health	Individual and group CBT for 3 months	14 CBT sessions by doctor and endocrinologist (manualization NS)	UC for 3 months	UC (regimen and therapist NS)	5	HbA1c, FBG, and SDS
(54)	598/598	56.4 ± 11.6	General	4.8 ± 3.9	Hospital (O)	Individual CBT^a^ and individual and group education for 7 months	CBT consisting of behavior modification and planning (number of sessions, therapist, and manualization NS), and individual and group education sessions (number of sessions and therapist NS)	UC for 7 months	UC and usual medication (regimen and therapist NS)	7	HbA1c and FBG
(55)	120/105	57.0 ± 8.5	General	5.3 ± 2.2	Hospital (I and O)	Individual CCT^a^ and health education for 6 months	12 non-directive counseling sessions with an emphasis on positive thinking by therapist, education sessions by doctor and therapist (number of sessions and manualization NS)	UC for 6 months	UC (regimen and therapist NS)	6	HbA1c and FBG
(56)	87/87	51.6 ± 8.0	General	5.3 ± 4.4	Hospital (I)	Individual CBT^a^, family therapy, and group education for 3 months	12–24 CBT sessions consisting of behavior modification, exercise therapy, psychological support, and family therapy, 12 education sessions; all by psychiatric attending physician (manualization NS)	UC for 3 months	UC and usual medication (regimen and therapist NS)	3	HbA1c, FBG, SAS, and SDS
(57)	108/108	50.9 ± 11.1	Depression	4.6 ± 3.4	Hospital (I and O)	Individual CBT for 6 weeks	CBT sessions consisting of relaxation training, music relaxation therapy, planning, and psychological support (number of sessions, therapist and manualization NS)	UC for 6 weeks	UC (regimen and therapist NS)	1.5	HbA1c, FBG, and HAM-D
(58)	86/86	53.0 ± 8.2	General	6.3 ± 2.2	Hospital (I)	Individual CBT for 1 month	8 CBT sessions consisting of stress management, behavior modification, planning, and psychological support by psychologist (manualization NS)	UC for 1 month	Usual nutrition, exercise, and medication (regimen and therapist NS)	1	SDS
(59)	94/94	53.1 ± 7.4	Nephropathy	NS	Hospital (I)	Individual and group CBT for 6 months	24 CBT sessions by psychologist (manualization NS)	UC for 6 months	UC consisting of education, glucose monitoring, nutrition, exercise, and monitoring of blood glucose, blood pressure, and lipids (regimen and therapist NS)	6	HbA1c, FBG, SAS, and SDS
(60)	438/438	51.6 ± NS	General	NS	Hospital (I)	Individual CBT for 1 month	CBT sessions by DM health professional (number of sessions and manualization NS)	UC for 1 month	Usual medication (regimen and therapist NS)	1	HbA1c, FBG, SAS, and SDS
(61)	82/80	56.8 ± 13.2	General	5.5 ± 4.7	Hospital (I and O)	Individual CBT^a^ for 1 month	8 CBT sessions consisting of relaxation training and psychological support (therapist and manualization NS)	UC for 1 month	UC (regimen and therapist NS)	1	HbA1c, FBG, and SCL-90
(62)	60/60	51.2 ± 11.7	General	3.3 ± 3.7	Hospital (I)	Individual CBT^a^ for 1 month	10 CBT sessions consisting of rational emotive therapy by diabetes specialist nurse (manualization NS)	UC and education for 1 month	UC and education (regimen and therapist NS)	1	SCL-90
(63)	118/118	NS (40–80)	Depression	6.0 ± 7.0	Hospital (I)	Individual CBT for 1 month	3–4 CBT sessions consisting of cognitive restructuring, relaxation training, and psychological support (therapist and manualization NS)	UC for 1 month	UC (regimen and therapist NS)	1	HbA1c, FBG, SAS, and SDS
(64)	72/72	53.1 ± 11.0	General	NS	Hospital (I)	Individual and group CBT and family therapy for 6 months	CBT sessions and family therapy by psychiatrist (number of sessions, therapist, and manualization NS)	UC for 6 months	Usual medication (regimen and therapist NS)	6	SCL-90 and DSQoL
(65)	63/63	63.1 (60–79)	Elderly (60+)	NS	Hospital (Military Psychiatric Hospital) (I)	Individual CBT^a^ and group education for 2 weeks	10 sessions of Qigong relaxation training, 10 sessions of music relaxation therapy, and 4 group education sessions all by doctor (manualization NS)	UC for 2 weeks	UC (regimen and therapist NS)	0.5	HAM-A and SCL-90
(66)	96/96	56.8 ± 3.9	General	7.5 (3–12)	Hospital (I)	Individual CCT^a^ and individual and group education for 3 months	Non-directive counseling sessions with an emphasis on improvement of self-esteem and positive thinking by psychologist (number of sessions and manualization NS), education sessions by DM specialist and nurse (number of sessions NS)	UC for 3 months	UC (regimen and therapist NS)	3	HbA1c, FBG, and SDS
(67)	67/67	44.5 ± 11.0	Depression and anxiety	4.6 ± 3.4	Hospital (I and O)	Individual and group CBT^a^ for 6 weeks	6 CBT sessions consisting of relaxation training, behavior modification, and psychological support by psychiatric attending physician (manualization NS)	UC for 6 weeks	Usual medication (regimen and therapist NS)	1.5	HbA1c, FBG, SAS, and SDS
(35)	70/70	60.5 ± 10.2	Depression	NS	Hospital (I)	Individual and group CBT^a^ for 2 months	8 CBT sessions (individual and group) consisting of relaxation training, emotional adjustment, and psychological support (therapist and manualization NS)	UC and education for 2 months	Usual nutrition, exercise and medication (regimen and therapist NS), 8 education sessions, and DM hotline by DM specialist nurse (regimen NS)	2	HbA1c, FBG, HAM-D, and DSQoL
(68)	93/93	53.6 ± 8.3	General	5.7 ± 3.7	Hospital (I)	Individual CBT^a^ and group education for 1 month	CBT consisting of emotional adjustment, relaxation training, exercise therapy (number of sessions and manualization NS), and 8 group education sessions; all by multidisciplinary team (endocrinologist, nurse, and dietitian)	UC for 1 month	Usual nutrition, exercise, and medication (regimen and therapist NS)	1	FBG, SAS, and SDS
(69)	300/300	NS (41–79)	General	6.0 ± NS	Hospital (I)	Individual CBT^a^ for 6 months	48 CBT sessions consisting of relaxation training, exercise therapy, emotional adjustment, behavior modification, and psychological support (therapist and manualization NS)	UC for 6 months	Usual medication by doctor (regimen NS)	6	HbA1c, FBG, and SCL-90
(70)	80/80	40.8 (22–60)	General	NS	Hospital (I)	Individual and group CBT^a^ for 3 months	12 CBT sessions consisting of morita therapy (individual and group), relaxation training, music relaxation therapy and psychological support by doctor and nurse (manualization NS), and education sessions by doctor and nurse (number of sessions NS)	UC for 3 months	UC consisting of clinical treatment and nursing care (regimen and therapist NS)	3	HbA1c, FBG, SAS, SDS, and DQoL
(71)	59/59	54.9 ± 8.4	General	NS (0.5–12)	Hospital (I)	Individual CBT^a^ and group education for 1 month	30 CBT sessions consisting of music relaxation therapy (therapist and manualization NS) and 2 group education sessions (therapist NS)	UC for 1 month	UC (regimen and therapist NS)	1	HbA1c, FBG, SAS, and SDS
(72)	300/300	52.6 ± 17.6	General	5.0 ± 3.6	Hospital (O)	Individual CBT^a^ and group education for 6 months	CBT sessions consisting of relaxation training and exercise therapy (number of sessions, therapist, and manualization NS), and education (number of sessions and therapist NS)	UC for 6 months	Usual medication (regimen and therapist NS)	6	HbA1c, FBG, SCL-90, and A-DQoL
(73)	80/80	54.8 ± 16.2	Low income	8.3 ± 6.7	Hospital (O)	Individual CBT for 3 months	12 CBT sessions consisting of morita therapy, emotional adjustment, and psychological support (therapist and manualization NS)	UC for 3 months	Usual nutrition, exercise, medication, and glucose monitoring (regimen and therapist NS)	3	HbA1c, FBG, and SDS
(74)	100/100	NS (20-70)	General	NS	Hospital (I)	Individual CBT^a^ for 1 month	8 CBT sessions consisting of behavior modification, relaxation training, cognitive restructuring, and psychological support by psychologist (manualization NS)	UC for 1 month	UC (regimen and therapist NS)	1	FBG, HAM-A, and HAM-D
(75)	68/68	58.0 ± 6.5	Depression	NS (3–5)	Hospital (I)	Individual CBT^a^ for 1 month	CBT sessions consisting of cognitive restructuring, emotional adjustment, and psychological support (number of sessions and manualization NS) by nurse	UC for 1 month	UC consisting of clinical treatment, psychological counseling, and education by nurse (regimen NS)	1	HbA1c, FBG, and SDS
(31)	86/86	50.2 ± 7.6	General	NS	Hospital (NS)	Individual CCT^a^ and individual education for 3 months	Non-directive counseling sessions with an emphasis on improvement of self-esteem (number of sessions, therapist and manualization NS)	UC for 3 months	Usual medication (regimen and therapist NS)	3	FBG, SAS, and SDS
(76)	80/71	60.4 ± 9.9	General	5.7 ± 3.1	Community health	Individual MI for 6 months	MI sessions by nurse (number of sessions and manualization NS)	UC and family education/glucose monitoring for 6 months	Usual nursing care and one family session every 1–3 months consisting of glucose monitoring and education by nurse	6	HbA1c
(77)	95/95	56.8 ± 12.5	General	NS	Hospital (I)	Group CBT and individual education for 2 weeks	5 group CBT consisting of relaxation training (therapist NS), and individual psychological support and education (number of sessions, therapist, and manualization NS)	UC for 2 weeks	UC (regimen and therapist NS)	0.5	FBG and DSQoL
(78)	200/200	52.4 ± 11.2	Depression	6.3 ± 4.2	Hospital (I and O)	Individual CBT for 1 month	8 CBT sessions consisting of behavior modification, relaxation training, psychological support, and exercise therapy (therapist and manualization NS)	UC for 1 month	Usual medication (regimen and therapist NS)	1	FBG and SDS
(79)	184/175	48.0 ± 8.0	General	4.0 ± 1.0	Hospital (O)	Individual and group CBT (duration NS)	20 CBT sessions – individual and group (therapist and manualization NS)	UC and education (duration NS)	UC and education (regimen and therapist NS)	NS	FBG and GQoLI-74
(80)	120/113	52.0 ± 12.0	General	NS (2–13)	Hospital (I)	Individual CBT^a^ for 2 months	8 CBT sessions consisting of morita therapy and relaxation training by psychologist (number of sessions and manualization NS)	UC for 2 months	Usual nutrition and medication (regimen and therapist NS)	2	FBG, SAS, and SDS
(32)	100/100	44.2 ± 6.8	General	4.6 ± 2.5	Hospital (I)	Individual CCT^a^ and family therapy for 1 month	Non-directive counseling sessions with an emphasis on improvement of self-esteem and positive thinking (number of sessions, therapist, and manualization NS) and family therapy (number of sessions and therapist NS)	UC for 1 month	Usual nutrition and medication (regimen and therapist NS)	1	HbA1c, FBG, SAS, and SDS
(81)	150/150	32.4 ± 12.0	General	5.3 ± 2.0	Hospital (I)	Individual CCT^a^, individual education and family therapy for 3–4 weeks	Non-directive counseling sessions with an emphasis on improvement of self-esteem and positive thinking (number of sessions and manualization NS) and family therapy (number of sessions NS); all by doctor and nurse	UC for 3–4 weeks	Usual nutrition, exercise, and medication (regimen and therapist NS)	0.75–1	HbA1c and FBG
(29)	120/120	NS (36–88)	General	NS	Hospital (I)	Individual MI for 3 sessions (duration NS)	3 MI sessions (therapist and manualization NS)	Usual education (duration NS)	Usual education consisting of group seminars and individual education focusing on nutrition, medication, exercise, and blood glucose monitoring (regimen and therapist NS)	NS	HbA1c and FBG
(82)	60/60	63 (50–75)	General	NS	Hospital (I)	Individual and group CBT^a^ for 1 month	8 CBT sessions consisting of relaxation training, music relaxation therapy, and emotional adjustment by doctor and nurse trained in psychology (manualization NS)	UC for 1 month	Usual nutrition, exercise, and medication by doctor and nurse (regimen NS)	1	FBG
(83)	120/106	55.9 (30–60)	General	5.5 (2–11)	Hospital (I and O)	Individual CBT^a^ and individual education for 6 months	Biofeedback assisted relaxation training (number of sessions NS), education (number of sessions NS), and 24 CBT sessions consisting of emotional adjustment and psychological support; all by psychologist (manualization NS)	UC for 6 months	Usual nutrition and medication (regimen and therapist NS)	6	HbA1c, FBG, and GQOLI-74
(84)	60/60	54.1 ± 11.9	General	NS (0.5–16)	NS	Individual CBT^a^ and medication for 1 month	8 CBT sessions consisting of cognitive restructuring, emotional adjustment, relaxation training, music relaxation therapy, and psychological support (medication regimen, therapist, and manualization NS)	Medication for 1 month	Medication (regimen and therapist NS)	1	FBG, SAS, and SDS
(85)^b^	80/75	66.9 ± 5.5	General	9.6 ± 4.7	Hospital (I)	Individual MI for 6 months	22 MI sessions by nurse (manualization NS)	Education for 6 months	6 group education sessions consisting of basic DM knowledge, nutrition, medication, exercise, glucose monitoring, and management of negative emotions (therapist NS)	6	HbA1c
(86)	128/87	Intervention: 53.96 ± 9.63 and control: 56.88 ± 8.87	General	Intervention: 4.8 ± 5.2 and control: 4.3 ± 4.2	Hospital (I)	Individual CBT^a^ for 1 month	8 sessions of biofeedback assisted relaxation training and music relaxation therapy (therapist and manualization NS)	UC for 1 month	UC (regimen and therapist NS)	8 months (1998 cohort) to 2 years (1996 cohort)	HbA1c and FBG
(27)^c^	67/56	54.0 ± 10.0	General	NS (0.5–16)	Hospital (I)	Individual CBT^a^ for 1 month	8 sessions of biofeedback assisted relaxation training and music relaxation therapy (therapist and manualization NS)	UC for 1 month	UC (regimen and therapist NS)	1	HbA1c and FBG
(87)	59/59	57.1 ± 9.6	General	4.8 ± 5.5	Hospital (I)	Individual CBT^a^ for 1 month	8–12 sessions of biofeedback assisted relaxation training and music relaxation therapy (therapist and manualization NS)	UC for 1 month	UC (regimen and therapist NS)	1	STAI

*^a^Categorized into a specific therapy by the psychological techniques utilized in intervention*.

*^b^Study not included in meta-analyses*.

*^c^Secondary article*.

*NS, not specified; I, inpatient; O, outpatient; CCT, client-centered therapy; CBT, cognitive behavioral therapy; MI, motivational interviewing; UC, usual care*.

*Outcome measure abbreviations: *Blood glucose concentration* – FBG, fasting blood glucose; *depression* – SDS, Zung self-rating depression scale; HAM-D, Hamilton depression rating scale; SCL-90 (DEP), symptom checklist-90 depression scale; *anxiety* – SAS, Zung self-rating anxiety scale; HAM-A, Hamilton anxiety rating scale; SCL-90 (ANX), symptom checklist-90 anxiety scale; STAI, state-trait anxiety inventory; *quality of life* – WHOQoL-BREF, WHO quality of life-BREF; DSQoL, disease-specific quality of life; DQoL, diabetes quality of life; A-DQoL, adjusted diabetes-specific quality of life; QOL-I, quality of life inventory; GQoLI-74, generic quality of life inventory-74*.

### Meta-Analyses

A summary of the estimated effects of the meta-analyses and heterogeneity are included in Table 2.

**Table 2:** **Summary of meta-analyses for psychological interventions vs. control condition**.

Outcomes	*K*	*N*	Effect estimate			Heterogeneity		
			SMD	95% CI	*Z* (*P*)	χ^2^	*P*	*I*^2^ (%)
**CBT vs. control**								
Glycated hemoglobin	20	2,900	−0.97	−1.37 to −0.57	4.72 (<0.0001)	430.87	<0.0001	96
Depression	27	3,084	−1.22	−1.51 to −0.94	8.39 (<0.0001)	331.61	<0.0001	92
Anxiety	21	2,479	−1.03	−1.26 to −0.80	8.91 (<0.0001)	130.53	<0.0001	85
Blood glucose concentration	28	3,802	−0.98	−1.25 to −0.72	7.20 (<0.0001)	373.18	<0.0001	93
Quality of life	5	722	−0.65	−1.35 to 0.06	1.80 (*P* = 0.07)	71.84	<0.0001	94
**MI vs. control**								
Glycated hemoglobin	3	251	−0.71	−1.00 to −0.43	4.88 (<0.0001)	2.43	0.30	18
**CCT vs. control**								
Glycated hemoglobin	4	451	−0.96	−1.97 to 0.05	1.86 (*P* = 0.06)	74.07	<0.0001	96
Depression	3	282	−0.86	−1.11 to −0.62	6.90 (<0.0001)	1.16	0.56	0
Blood glucose concentration	5	537	−1.31	−2.42 to −0.20	2.31 (*P* = 0.02)	130.69	<0.0001	97

*CBT, cognitive behavioral therapy; MI, motivational interviewing; CCT, client-centered therapy; *K*, number of studies; *N*, number of participants; SMD, standardized mean difference; CI, confidence interval*.

#### Cognitive Behavioral Therapy

**Figure 2:**
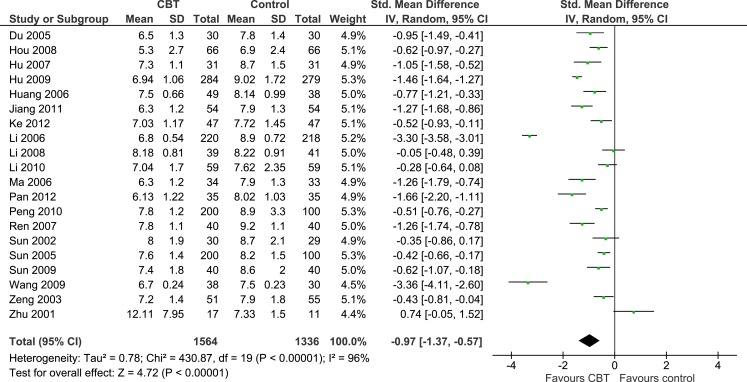
**Meta-analysis illustrating the standardized effects of cognitive behavioral therapy (CBT) vs. control for glycated hemoglobin**.

Twenty RCTs (*n* = 2,900) were included in the meta-analysis comparing CBT with a control group for glycated hemoglobin and results indicated that CBT was more beneficial than the control condition (SMD = −0.97, 95% CI: −1.37 to −0.57) (Figure 2). This corresponded to an effect size of −1.56% in absolute units. The χ^2^ test for heterogeneity was significant, with considerable heterogeneity (χ^2^ = 430.87, *P* < 0.0001, *I*^2^ = 96%). Various subgroup analyses were conducted to explore this heterogeneity. While there were slight variations in the effect sizes, no significant differences were found between the following subgroups: clinical subgroup (*P* = 0.32), modality of therapy (*P* = 0.50), timing of glycated hemoglobin outcome assessment (*P* = 0.16), and classification of treatment type (*P* = 0.68).

Twenty-seven RCTs (*n* = 3,084) were included in the meta-analysis comparing CBT with a control group for depression and results indicated that CBT was more beneficial than the control condition (SMD = −1.22, 95% CI: −1.51 to −0.94) (Figure S1 in Supplementary Material). The χ^2^ test for heterogeneity was significant, with considerable heterogeneity (χ^2^ = 331.61, *P* < 0.0001, *I*^2^ = 92%). Various subgroup analyses were conducted to explore this heterogeneity. While there were slight variations in the effect sizes, no significant differences were found between the following subgroups: clinical subgroup (*P* = 0.99), modality of therapy (*P* = 0.23), and classification of treatment type (*P* = 0.39).

Twenty-one RCTs (*n* = 2,479) were included in the meta-analysis comparing CBT with a control group for anxiety and results indicated that CBT was more beneficial than the control condition (SMD = −1.03, 95% CI: −1.26 to −0.80) (Figure S2 in Supplementary Material). The χ^2^ test for heterogeneity was significant, with substantial to considerable heterogeneity (χ^2^ = 130.53, *P* < 0·0001, *I*^2^ = 85%). Various subgroup analyses were conducted to explore this heterogeneity. While there were slight variations in the effect sizes, no significant differences were found between the following subgroups: clinical subgroup (*P* = 0.37), modality of therapy (*P* = 0.44), and classification of treatment type (*P* = 0·41).

Twenty-eight studies (*n* = 3,802) were included in the meta-analysis comparing CBT with a control group for blood glucose concentration and results indicated that CBT was more beneficial than the control condition (SMD = −0.98, 95% CI: −1.25 to −0.72) (Figure S3 in Supplementary Material). The χ^2^ test for heterogeneity was significant, with considerable heterogeneity (χ^2^ = 373.18, *P* < 0.0001, *I*^2^ = 93%). Various subgroup analyses were conducted to explore this heterogeneity. While there were slight variations in the effect sizes, no significant differences were found between the following subgroups: clinical subgroup (*P* = 0.48), modality of therapy (*P* = 0.35), and classification of treatment type (*P* = 0.81).

Five studies (*n* = 722) were included in the meta-analysis comparing CBT with a control group for overall QOL and results indicated that the difference between CBT and the control condition was not significant (SMD = −0.65, 95% CI: −1.35 to 0.06) (Figure S4 in Supplementary Material). The χ^2^ test for heterogeneity was significant, with considerable heterogeneity (χ^2^ = 71.84, *P* < 0·0001, *I*^2^ = 94%). There were insufficient studies to conduct subgroup analyses to explore this heterogeneity.

Funnel plots and Egger’s tests were conducted for all CBT outcomes with the exception of QOL, for which there were insufficient studies (*k* = 5). No evidence of publication bias was found for two of the outcomes, glycated hemoglobin (*P* = 0.608), and blood glucose concentration (*P* = 0.873). However, publication bias was indicated for the CBT outcomes of depression (*P* = 0.001) and anxiety (*P* = 0.007) (Funnel plots and Egger’s test not shown – see Supplementary Material).

#### Motivational Interviewing

**Figure 3:**
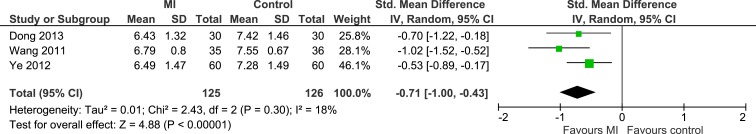
**Meta-analysis illustrating the standardized effects of motivational interviewing (MI) vs. control for glycated hemoglobin**.

Three studies (*n* = 251 participants) were included in the meta-analysis comparing MI with a control group for glycated hemoglobin and results indicated that MI was more beneficial than the control condition (SMD = −0.71, 95% CI: −1.00 to −0.43) (Figure 3). This corresponded to an effect size of −0.92% in absolute units. The χ^2^ test for heterogeneity was not significant, with heterogeneity considered not important (χ^2^ = 2.43, *P* = 0.30, *I*^2^ = 18%).

There were insufficient studies to conduct a meta-analysis comparing MI with a control for the additional outcomes. Qualitatively, two studies assessed blood glucose concentration, and both found significant improvements (*P* > 0.05) favoring MI compared with the control (28, 29). Additionally, two studies assessed QOL, and both found significant improvements (*P* > 0.05) favoring MI compared with the control (28, 30). No studies utilizing MI assessed depression or anxiety.

#### Client-Centered Therapy

Four studies (*n* = 451 participants) were included in the ­meta-analysis comparing CCT with a control group for glycated hemoglobin, and results indicated that the difference between CCT and the control condition was not significant (SMD = −0.96, 95% CI: −1.97 to 0.05) (Figure S5 in Supplementary Material). The χ^2^ test for heterogeneity was significant, with considerable heterogeneity (χ^2^ = 74.07, *P* < 0.0001, *I*^2^ = 96%).

Three studies (*n* = 282 participants) were included in the meta-analysis comparing CCT with a control group for depression and results indicated that CCT was more beneficial than the control condition (SMD = −0.86, 95% CI: −1.11 to −0.62) (Figure S6 in Supplementary Material). The χ^2^ test for heterogeneity was not significant, with heterogeneity considered not important (χ^2^ = 1.16, *P* = 0.56, *I*^2^ = 0%).

Five studies (*n* = 537 participants) were included in the meta-analysis comparing CCT with a control group for blood glucose concentration. Results indicated that CCT was more beneficial than the control condition (SMD = −1.31, 95% CI: −2.42 to −0.20) (Figure S7 in Supplementary Material). The χ^2^ test for heterogeneity was significant, with considerable heterogeneity (χ^2^ = 130.69, *P* < 0.0001, *I*^2^ = 97%).

There were insufficient studies to conduct subgroup analyses for any of the CCT comparisons and insufficient studies to conduct a meta-analysis comparing CCT with a control for the anxiety. Qualitatively, two RCTs assessed anxiety and both found significant improvements (*P* > 0·05) favoring CCT compared with the control (31, 32). No RCTs utilizing CCT assessed overall QOL.

### Risk of Bias Assessment

Table 3 summarizes the risk of bias assessment of the included studies. Eight RCTs clearly described an adequate random sequence generation (e.g., random number table) and were classified as low risk of bias. Four RCTs were classified as high risk of bias, as the sequence generation procedure used included a non-random component (e.g., allocation based on admission date). Thirty-five RCTs did not provide enough information on the randomization procedure and were classified as unclear.

**Table 3:** **Risk of bias summary**.

References	Random sequence generation (*selection bias*)	Allocation concealment (*selection bias*)	Blinding of outcome assessment (*detection bias*)	Incomplete outcome data (*attrition bias*)	Selective reporting (*reporting bias*)
(47)	?	?	?	+	?
(30)	?	?	?	?	−
(48)	?	?	?	+	?
(49)	+	?	?	+	?
(50)	?	?	?	+	?
(28)	?	?	?	+	?
(51)	?	?	?	+	?
(52)	?	?	?	+	?
(53)	?	?	?	?	−
(54)	?	?	?	?	?
(55)	?	?	?	?	?
(56)	+	?	?	?	?
(57)	?	?	?	?	?
(58)	?	?	?	+	?
(59)	?	?	?	+	?
(60)	?	?	?	?	?
(61)	?	?	?	?	?
(62)	?	?	?	+	?
(63)	+	?	?	+	?
(64)	?	?	?	?	?
(65)	?	?	?	?	?
(66)	?	?	?	+	?
(67)	+	?	?	?	?
(35)	?	?	?	+	?
(68)	−	?	?	+	−
(69)	?	?	?	+	?
(70)	?	?	?	+	−
(71)	?	?	?	?	?
(72)	−	?	?	?	?
(73)	?	?	?	+	?
(74)	?	?	?	+	?
(75)	+	?	?	+	?
(31)	?	?	?	+	?
(76)	+	?	?	?	?
(77)	?	?	?	?	?
(78)	?	?	?	+	?
(79)	?	?	?	?	?
(80)	−	?	?	?	?
(32)	?	?	?	+	?
(81)	?	?	?	+	?
(29)	+	?	?	+	?
(82)	?	?	?	+	?
(83)	−	?	?	?	−
(84)	?	?	?	+	−
(85)	+	?	?	?	−
(27, 86)	?	?	?	?	?
(87)	?	?	?	+	?

*Low risk of bias (+); high risk of bias (–); and unclear risk of bias (?)*.

All RCTs were classified as having an unclear risk of bias for allocation concealment and blinding of outcome assessors due to insufficient information.

Twenty-seven RCTs used an intention-to-treat approach and were classified as low risk of bias for incomplete outcome data. Twenty RCTs did not provide information on how missing data was handled and were classified as unclear.

Forty RCTs were classified as unclear for selective outcome reporting due to no protocols being available to determine if all outcome data collected was reported. Furthermore, it was difficult to ascertain through the published reports that all of the expected outcomes were reported (24). Seven RCTs were classified as having a high risk of bias as they did not report on all prespecified outcomes or time points, or the information provided did not allow inclusion in the meta-analysis.

## Discussion

This systematic review is the first to assess the effectiveness of psychological interventions in improving T2DM-related outcomes in China. By systematically searching Chinese-language databases in addition to international (English language) databases, 40 additional RCTs were identified from that of previous reviews (15, 16). Furthermore, this review is the first to differentiate between the types of psychological interventions commonly utilized in the management of patients with T2DM. This enabled a more comprehensive and rigorous review and added strength to the meta-analyses conducted.

With regard to the primary outcome measure, glycated hemoglobin, results indicate that CBT and MI were more effective than the control condition, endorsing the use of the aforementioned psychological therapies for the improvement of glycemic control among patients with T2DM. The observed effect sizes in glycated hemoglobin were −0.97 (CBT) and −0.71 (MI), which resulted in absolute reductions in HbA1c of −1.56% (CBT) and −0.92% (MI). Based on the UK Prospective Diabetes Study (33), these effects are enough to reduce the risk of development and progression of T2DM microvascular complications (15). Furthermore, these effect sizes and absolute differences are consistent, and higher, than previous meta-analyses of predominantly English-language literature which also found psychological interventions to be effective in improving glycemic control [effect sizes ranging from −0.29 (17) to −0.32 (15, 16); and absolute differences in HbA1c ranging from −0.54% (16) to −0.76% (15)].

Separate meta-analyses found both CBT and CCT to be associated with reductions in depression, and CBT with reductions in anxiety. Previous meta-analyses have similarly associated psychological interventions with improvements in psychological status (15, 16) and mental health (17); however, these studies combined depression, anxiety, wellbeing, and QOL into one outcome. Consequently, the differential effect of psychological interventions on each outcome has not previously been noted and is a feature unique to this review.

Improvement in blood glucose concentration was also observed in the meta-analysis for CBT. Only one previous meta-analysis has included blood glucose concentration as an outcome measure and in contrast found the effect size to be not significant (15).

In contrast to all other CBT meta-analyses, the observed effect of CBT on overall QoL was not significant. While this may partly be attributed to few included studies (*k* = 5), previous research has found that the effect of depression on QoL is greater than the effect of DM on QoL (34). Only one included study (35) was comprised of T2DM patients with comorbid depression, with the remainder of studies conducted in general T2DM populations. It is possible that had more studies included T2DM patients with comorbid depression, larger effect sizes may have been observed.

While this review adhered to the PRISMA guidelines (20) and used systematic and rigorous methods based on the Cochrane Collaborations’ recommendations (24), various limitations of the included studies suggest caution when interpreting the results. Validity, as assessed through the risk of bias (22), was unclear for the majority of included studies, predominantly due to the lack of reporting of vital information, such as allocation concealment and blinding of outcome assessors. Most studies did not follow recognized reporting standards, such as the CONSORT statement (36), and consequently, information required for an adequate risk of bias assessment was largely missing.

Additionally, publication bias was evident for two of the CBT outcomes (depression and anxiety), and the level of heterogeneity was considerable for all outcomes utilizing CBT and CCT (with the exception of depression for CCT). While this heterogeneity could not be explained despite the various subgroup analyses conducted, the heterogeneity is most probably caused by a combination of several confounding factors (e.g., clinical and methodological diversity). Furthermore, the heterogeneity observed in CBT and CCT is potentially associated with the wide range of techniques that encompassed CBT, as well as the unstructured, non-directive nature of CCT. In comparison, MI, while non-directive, is a specific technique that is focused and goal-oriented with less opportunity for variation between RCTs.

It is also worth noting that none of the included studies reported using manualized interventions or assessed treatment integrity of the psychological therapies utilized. This review tightly limited eligibility to studies comparing psychological therapies to a control condition; however, it is difficult to ascertain whether the effective component of each intervention was the psychological therapy itself, or if it was a combination of increased monitoring of individuals in each of the intervention groups. The majority of patients with T2DM in China do not regularly monitor their blood glucose concentration or attend regular appointments specifically to manage their T2DM (37, 38). Therefore, the effect size attributed to each psychological therapy may have been overestimated due to increased monitoring and attention to the health care of individuals in each intervention group rather than due to the specific psychological therapy.

The utilization of psychological therapies for behavior change in chronic disease management is a relatively new field in China, and this was reflected by the publication date of included studies (only *k* = 1 published before 2000). With the combination of China’s increasing T2DM burden, there is a huge potential for psychological interventions to be conducted in China. In particular, this review highlights that interventions are required that utilize specific psychological therapies (i.e., manualized CBT) and not just components of recognized therapies (i.e., relaxation training as a component of CBT). Additionally, more interventions utilizing MI and CCT would enable a more thorough assessment of outcomes of interest and would allow for heterogeneity and publication bias to be adequately investigated. In line with China’s current primary health care reform which is aiming to shift T2DM management from a hospital-based model, toward delivery in primary health care settings (39), there is a pressing need for interventions to be conducted at the community health level that utilize existing resources. In this study, the population group most frequently sampled were hospital inpatients (*k* = 38), and only three of the included studies were conducted in a community health setting (one utilizing CBT and two utilizing MI). Previous research has observed differences in anthropometric, biochemical, and cardiovascular risk profiles between inpatients and outpatients (40). As such, generalizability of these findings to the community health setting is limited. Despite the recency of published articles, there were no interventions that utilized technology in the delivery of their intervention, and this is also an opportunity for further research. Finally, interventions of future RCTs in China should include longer follow-up periods and report the interventions according to the CONSORT statement (36) in order to meet international standards, to enable adequate risk of bias assessments to be performed, and to increase the rigor of future reviews.

With regard to recommendations for future systematic reviews and meta-analyses, the additional 40 RCTs identified in this review highlights the importance of intentionally utilizing language/country specific databases in future search strategies. The inclusion of more non-English publications in systematic reviews limits the potential for language bias and increases the precision and statistical power of meta-analysis estimates (41). When relevant, the use of regional databases is promoted by the Cochrane Collaboration (24), and Chinese biomedical databases are a potential resource that should be considered.

In conclusion, this systematic review and meta-analyses demonstrated that psychological interventions, namely, CBT, MI, and CCT, are effective in improving certain T2DM related outcomes in China. Considerable levels of heterogeneity and unclear risk of bias associated with the most included RCTs warrant caution when interpreting results. If China is to address the health issues associated with its burgeoning T2DM population and delay the progression of T2DM-related outcomes, psychological interventions are promising tools and should be utilized in the future.

## Author Contributions

AC, SM, CB, and ST contributed to the study conception and design. AC, SM, and SL did the systematic literature search. AC, SM, SL, and HY selected studies for inclusion, and SL and HY extracted the data. AC and SM assessed risk of bias of included studies. AC, SM, and JE performed the statistical analysis, interpreted data, and wrote the first draft of the manuscript. All authors critically revised the manuscript and approved the final version. All authors, internal and external, had full access to the study data and take responsibility for the integrity of the data and the accuracy of the data analysis. All authors have approved the final version of the manuscript.

## Conflict of Interest Statement

The authors of this manuscript declare no conflict of interest. There have been no payments or services for the work of this manuscript. There are no financial relationships with any entities that may have influenced the work of this manuscript. There are no declarations for patent or copyright for work related to this manuscript.

## References

[1] American Diabetes Association. Diagnosis and classification of diabetes mellitus. Diabetes Care (2010) 33(Suppl 1):S62–9. 10.2337/dc10-S062 20042775 PMC2797383

[2] American Diabetes Association. Standards of medical care in diabetes – 2013. Diabetes Care (2013) 36(Suppl 1):S11–66. 10.2337/dc13-S011 23264422 PMC3537269

[3] Scottish Intercollegiate Guidelines Network (SIGN). Management of Diabetes. A National Clinical Guideline. Edinburgh: Scottish Intercollegiate Guidelines Network (SIGN) (2010).

[4] Royal Australian College of General Practitioners. Diabetes Management in General Practice: Guidelines for Type 2 Diabetes. Melbourne: Royal Australian College of General Practitioners and Diabetes Australia. General Practice Management of Type 2 Diabetes – 2014–15 (2014).

[5] Canadian Diabetes Association Clinical Practice Guidelines Expert Committee. Clinical practice guidelines for the prevention and management of diabetes in Canada. Can J Diabetes (2013) 37(Suppl 1):S1–216. 10.1016/j.jcjd.2013.01.009 24070926

[6] Meurs M Roest AM Wolffenbuttel BH Stolk RP de Jonge P Rosmalen JG. Association of depressive and anxiety disorders with diagnosed versus undiagnosed diabetes: an epidemiological study of 90,686 participants. Psychosom Med (2015). 10.1097/psy.0000000000000255 26452174

[7] Collins MM Corcoran P Perry IJ. Anxiety and depression symptoms in patients with diabetes. Diabet Med (2009) 26(2):153–61. 10.1111/j.1464-5491.2008.02648.x 19236618

[8] Gonzalez JS Peyrot M McCarl LA Collins EM Serpa L Mimiaga MJ Depression and diabetes treatment nonadherence: a meta-analysis. Diabetes Care (2008) 31(12):2398–403. 10.2337/dc08-1341 19033420 PMC2584202

[9] Pan C Diabetes care in China: meeting the challenge. Diabetes Voice (2005) 50(2):9–12. 16300163

[10] International Diabetes Federation. IDF Diabetes Atlas (2014 Update). Brussels: International Diabetes Federation (2014).

[11] Yang H Thomas SA Browning CJ Chronic disease management. 1 ed In: Li Z Zhang Y Yang H, editors. Community Health Services Management. Beijing: People’s Military Medicine Publisher (2010). p. 65–97.

[12] Bhattacharyya O Delu Y Wong ST Bowen C Evolution of primary care in China 1997–2009. Health Policy (2011) 100(2–3):174–80. 10.1016/j.healthpol.2010.11.005 21145123

[13] Browne D The long march to primary health care in China: from collectivism to market economics. Public Health (2001) 115(1):2–3. 10.1038/sj.ph.1900742 11402345

[14] Gao X Jackson T Chen H Liu Y Wang R Qian M There is a long way to go: a nationwide survey of professional training for mental health practitioners in China. Health Policy (2010) 95(1):74–81. 10.1016/j.healthpol.2009.11.004 19962778

[15] Ismail K Winkley K Rabe-Hesketh S. Systematic review and meta-analysis of randomised controlled trials of psychological interventions to improve glycaemic control in patients with type 2 diabetes. Lancet (2004) 363(9421):1589–97. 10.1016/S0140-6736(04)16202-8 15145632

[16] Alam R Sturt J Lall R Winkley K. An updated meta-analysis to assess the effectiveness of psychological interventions delivered by psychological specialists and generalist clinicians on glycaemic control and on psychological status. Patient Educ Couns (2009) 75(1):25–36. 10.1016/j.pec.2008.08.026 19084368

[17] Harkness E MacDonald W Valderas J Coventry P Gask L Bower P. Identifying psychosocial interventions that improve both physical and mental health in patients with diabetes: a systematic review and meta-analysis. Diabetes Care (2010) 33(4):926–30. 10.2337/dc09-1519 20351228 PMC2845054

[18] Fung IC. Chinese journals: a guide for epidemiologists. Emerg Themes Epidemiol (2008) 5:20. 10.1186/1742-7622-5-20 18826604 PMC2648956

[19] Wu T Li Y Bian Z Liu G Moher D. Randomized trials published in some Chinese journals: how many are randomized? Trials (2009) 10(1):46. 10.1186/1745-6215-10-46 19573242 PMC2716312

[20] Moher D Liberati A Tetzlaff J Altman DG Preferred reporting items for systematic reviews and meta-analyses: the PRISMA statement. Br Med J (2009) 339:b2535 10.1136/bmj.b2535 19622551 PMC2714657

[21] Chinese Diabetes Society. China’s prevention and treatment guideline for type II diabetes mellitus (2010 edition). Chin J Front Med Sci (2011) 3(6):54–109.

[22] Higgins JPT Altman DG Assessing risk of bias in included studies. In: Higgins JPT Green S, editors. Cochrane Handbook for Systematic Reviews of Interventions. Chichester: John Wiley & Sons, Ltd (2008). p. 187–241.

[23] The Cochrane Collaboration. Review Manager (RevMan). 5.3.5 ed Copenhagen: The Nordic Cochrane Centre (2014).

[24] Higgins J Green S Cochrane Handbook for Systematic Reviews of Interventions. Version 5.1.0. Chichester: The Cochrane Collaboration (2011).

[25] Dickersin K Berlin JA Meta-analysis: state-of-the-science. Epidemiol Rev (1992) 14(1):154–76. 1289110 10.1093/oxfordjournals.epirev.a036084

[26] Egger M Davey Smith G Schneider M Minder C Bias in meta-analysis detected by a simple, graphical test. Br Med J (1997) 315(7109):629–34. 10.1136/bmj.315.7109.629 9310563 PMC2127453

[27] Zhu XZ Dong X Yao S The effect of biofeedback assisted relaxation training on glucose metabolism in patients with type 2 diabetes. Chin J Diabetes (1998) 6(3):167–70.

[28] Dong M Application of motivational interviewing to exercise therapy in type 2 diabetic patients. J Qilu Nurs (2013) 19(15):12–4. 10.3969/j.issn.1006-7256.2013.15.005

[29] Ye Q Zheng L Affect by motivational interviewing to type 2 diabetes patients’ behaviour change. Chin J Prim Med Pharm (2012) 19(17):2713–4. 10.3760/cma.j.issn.1008-6706.2012.17.110

[30] Browning C Thomas S Yang H Chapman A Zhang T Li Z The happy life club™ (5): improving quality of life of patients of type 2 diabetes mellitus. J Chin Gen Pract (2011) 14(13):1397–401. 10.3969/j.issn.1007-9572.2011.13.001

[31] Wang T The effect of psychological intervention on depression, anxiety and glucose control of patients with diabetes. Chin Commun Doctors (2010) 12(6):132 10.3969/j.issn.1007-614x.2010.06.161

[32] Yang D Ma J Effect of psychological behavioral intervention on affect disorder and glucose metobolism of type 2 diabetics. J Nurs Train (2008) 23(12):1136–8. 10.3969/j.issn.1002-6975.2008.12.039

[33] UK Prospective Diabetes Study Group. Intensive blood-glucose control with sulphonylureas or insulin compared with conventional treatment and risk of complications in patients with type 2 diabetes (UKPDS 33). Lancet (1998) 352(9131):837–53. 10.1016/S0140-6736(98)07019-6 9742976

[34] Goldney RD Phillips PJ Fisher LJ Wilson DH. Diabetes, depression, and quality of life: a population study. Diabetes Care (2004) 27(5):1066–70. 10.2337/diacare.27.5.1066 15111522

[35] Pan S Effect of psychological nursing care on type 2 diabetes patients with depression disorder. J Med Theor Pract (2012) 25(22):2839–41. 10.3969/j.issn.1001-7585.2012.22.079

[36] Schulz KF Altman DG Moher D CONSORT 2010 statement: updated guidelines for reporting parallel group randomised trials. BMJ (2010) 340:c332 10.1136/bmj.c332 20332509 PMC2844940

[37] Pan C. Diabetes care in China: meeting the challenge. World Hosp Health Serv (2005) 50(2):9–12. 16300163

[38] Xu Y Toobert D Savage C Pan W Whitmer K. Factors influencing diabetes self-management in Chinese people with type 2 diabetes. Res Nurs Health (2008) 31(6):613–25. 10.1002/nur.20293 18613066

[39] Liu Q Wang B Kong Y Cheng KK China’s primary health-care reform. Lancet (2011) 377(9783):2064–6. 10.1016/S0140-6736(11)60167-0 21453962

[40] Margari F Zagaria G Lozupone M Minerva F Pisani R Palasciano G Metabolic syndrome: differences between psychiatric and internal medicine patients. Int J Psychiatry Med (2013) 45(3):203–26. 10.2190/PM.45.3.a 24066405

[41] Cohen JF Korevaar DA Wang J Spijker R Bossuyt PM. Should we search Chinese biomedical databases when performing systematic reviews? Syst Rev (2015) 4:23. 10.1186/s13643-015-0017-3 25874584 PMC4374381

[42] Beck JS Beck AT Cognitive Behavior Therapy: Basics and Beyond. 2 ed New York, NY: Guilford Publications (2011).

[43] Miller WR Rollnick S Motivational Interviewing: Helping People Change. Third ed New York, NY: Guilford Press (2012).

[44] Rogers CR Client-Centered Therapy: Its Current Practice, Implications and Theory. London: Constable (1951).

[45] Messer SB What makes brief psychodynamic therapy time efficient. Clin Psychol SciPract (2001) 8(1):5–22. 10.1093/clipsy.8.1.5

[46] Stuart S Robertson M Interpersonal Psychotherapy: A Clinician’s Guide. 2 ed Boca Raton, FL: Taylor & Francis (2012).

[47] Bao M Observation of psychological nursing care of elder diabetes patients with depression disorder. J Med Theor Pract (2013) 26(5):673–5. 10.3969/j.issn.1001-7585.2013.05.082

[48] Chen F Chen Z Influence of psychological interference on the quality of life for type 2 diabetes patients. Hebei Med (2006) 12(1):3–6. 10.3969/j.issn.1006-6233.2006.01.002

[49] Chen C Li Z The effect of psychological therapy accommodates emotional and psychological barriers of diabetic. Guide Chin Med (2013) 11(27):26–7. 10.3969/j.issn.1671-8194.2013.27.014

[50] Dong X Dai W Bian S Liu S Wang S Effect of rational-emotion therapy on emotion status and self-management behaviors of elder type 2 diabetics. Chin J Gerontol (2012) 32(1):157–8. 10.3969/j.issn.1005-9202.2012.01.073

[51] Du W Zhang Q Zhang Z Effects of psycho-intervention on patients with type 2 diabetes accompanying depression. J Clin Psychosom Dis (2005) 11(4):341–2. 10.3969/j.issn.1672-187X.2005.04.022

[52] Hou J Liu S Wang X Effect of systematic psychological intervention on anxiety and depression of patients with type 2 diabetes. Chin J Rehabil (2008) 23(5):349 10.3870/zgkf.2008.05.028

[53] Hu Y Zhang J Effect of community comprehensive psychological and behavioral intervention on glucose metabolism of type 2 diabetes patients complicated with depression. J Chin Gen Pract (2007) 10(16):1383–6. 10.3969/j.issn.1007-9572.2007.16.033

[54] Hu Y Xu S Liu C The effect of health education on the blood glucose controlling target in patients with type 2 diabetes mellitus. Chin J Prev Contr Chron Non Commun Dis (2009) 17(2):188–9.

[55] Huang F Ye J Huang F The effect of mental intervention on type 2 diabetics: a randomized controlled study. Chin J Clin Rehabil (2004) 8(27):5795–7. 10.3321/j.issn:1673-8225.2004.27.025

[56] Huang Z Tao J Effects of integrative psychological and behavior intervention on the patients with type 2 diabetes. J Nurs Sci (2006) 21(7):7–9. 10.3969/j.issn.1001-4152.2006.07.003

[57] Jiang H Effects of systemic psycholosical intervention on symptom and life quality of type 2 diabetes with depression. China J Health Psychol (2011) 19(1):34–6.

[58] Jin S Chen X Mu J Chen Z Effect of psychotherapy on depression with type II diabetes patients. China J Health Psychol (2007) 15(1):94–5. 10.3969/j.issn.1005-1252.2007.01.030

[59] Ke X Wang Q Effect of psychological treatment on type 2 diabetic nephropathy. J Huangshi Inst Technol (2012) 28(1):54–6. 10.3969/j.issn.1008-8245.2012.01.014

[60] Li Z Lu G Peng A Effect on mental therapy in the patients with type 2 diabetes. China J Health Psychol (2006) 14(2):212–4. 10.3969/j.issn.1005-1252.2006.02.026

[61] Li Y Tang S Chen W Effects of comprehensive psychological intervention on emotion and carbohydrate metabolism in patients with type 2 diabetes mellitus. J Chin Gen Pract (2008) 6(12):1279–80. 10.3969/j.issn.1674-4152.2008.12.040

[62] Li J Wang M Wang R Zhang J Zheng Y Long J Effect of self-management intervention on psychology and behavior of diabetic patients. J Nurs Sci (2009) 24(13):72–4. 10.3870/hlxzz.2009.13.072

[63] Li M Jiang D Wang J Effect of psychological cognitive nursing of type 2 diabetics with depression disorder. Today Nurse (2010) 8:83–4. 10.3969/j.issn.1006-6411.2010.08.054

[64] Li M Wang X Ma Y Wan A Effects of psychological intervention on preliminary diagostic type 2 diabetes’ survival quality and hospital stay. Mod Prev Med (2011) 38(8):1473–5.

[65] Liu T Tian J Yan J Effect of psychological intervention on the curative effect of the senile diabetes. Chin J Behav Med Sci (2000) 5:339–40. 10.3760/cma.j.issn.1674-6554.2000.05.008

[66] Liu Y Liu L Systemic education and psychology interference on depression and carbohydrate metabolism of the patients with type 2 diabetes. J Nurs Sci (2003) 18(10):727–9. 10.3969/j.issn.1001-4152.2003.10.002

[67] Ma Z Li M Wang C Effects of comprehensive psycho-intervention on life quality and carbohydrate metabolism in patients with type 2 diabetes mellitus. Chin J Clin Rehabil (2006) 10(30):15–7. 10.3321/j.issn:1673-8225.2006.30.005

[68] Peng M Ou X Wang X The influence of psychological intervention on negative emotion and glucose level control of patients with type 2 diabetes. Int J Nurs (2009) 28(1):102–5.

[69] Peng Y Jiang Y Zhou H Effect of personal psychological nursing on medication treatment of type 2 diabetes. Pract Cardio Cerebr Pulm Vasc Dis (2010) 18(6):731–2. 10.3969/j.issn.1008-5971.2010.06.018

[70] Ren M Wei H Gao G Application of morita therapy and relaxation therapy in diabetes patients’ health education. J Qilu Nurs (2007) 7:96–7. 10.3969/j.issn.1006-7256.2007.07.101

[71] Sun F Fang R Cheng J A control study of effect of comprehensive psychological intervention for patients with type 2 diabetes. Health Psychol J (2002) 10(6):465–6. 10.3969/j.issn.1005-1252.2002.06.039

[72] Sun B Ban B Sun H Effects of diabetes education and psychological intervention on comprehensive treatment of type 2 diabetes. Chin J Clin Psychol (2005) 13(4):483–5. 10.3969/j.issn.1005-3611.2005.04.039

[73] Sun H Zhang Q Effects of psychological interventions on depression and glucose metabolism of low-income people with type 2 diabetes. China Clin Pract Med (2009) 3(11):96–7. 10.3760/cma.j.issn1673-8799.2009.11.62

[74] Wang X Hu W Effects of mental intervention on anxiety depression emotions of type 2 diabetics. J Clin Psychosom Dis (2008) 14(5):418–9. 10.3969/j.issn.1672-187X.2008.05.015

[75] Wang P Han P Zhang Y Wang Z Effect of nursing intervention on glucose and lipid metabolism in diabetic patients with depression. Chin J Nurs Educ (2009) 6(10):470–2. 10.3761/j.issn.1672-9234.2009.10.015

[76] Wang X Zhang J Wang F Li Z Meijing. Application of motivational interviewing in community of type 2 diabetes patients’ health education. Anhui Med J (2011) 32(12):2066–8. 10.3969/j.issn.1000-0399.2011.12.052

[77] Wei S Guo W Liang Z Yan D Study of mental health educating in hospital effecting on type 2 diabetes mellitus patience’s quality of life and glycometabolism. J Pract Med Tech (2008) 15(10):1236–8. 10.3969/j.issn.1671-5098.2008.10.007

[78] Wu G Huang P The effect of psychological intervention on treatment of patients with diabetes. China Mod Doctor (2008) 46(10):62–3. 10.3969/j.issn.1673-9701.2008.10.034

[79] Xiao L Liang T Wei D The effect of cognitive behavioral therapy on quality of life in type 2 diabetes. Chin J Behav Med Sci (2006) 15(7):591–2. 10.3760/cma.j.issn.1674-6554.2006.07.006

[80] Yan Q Li J Influence of psychological counseling on type 2 diabetic patients’ blood sugar level and emotion. W China Med J (2011) 26(9):1306–8.

[81] Yang L Zhang S Mao Y Wei J Effect of psychological nursing care on disease control of patients with diabetes. Shanxi Med J (2009) 38(Suppl):90–1. 10.3969/j.issn.0253-9926.2009.z1.060

[82] Yi L Zhou C The influence of mental intervention on blood sugar in type 2 diabetes mellitus. Med J Qilu (2012) 27(6):529–30. 10.3969/j.issn.1008-0341.2012.06.027

[83] Zeng Z Ma L Tang L Luo G Zhou L Comprehensive intervention on diabetes millitus. Chin Ment Health J (2003) 17(4):253–5. 10.3321/j.issn:1000-6729.2003.04.012

[84] Zhang Z Shan B Wang X Ma A Leng Y The effect of psychological intervention on negative emotion and glucose control of patients with diabetes. Chin J Behav Med Sci (2001) 10(5):452–513. 10.3760/cma.j.issn.1674-6554.2001.05.025

[85] Zhang Y Zhao Y Xu L Hu H Hu S Application of motivational interviewing to diabetic education for elderly patients with type 2 diabetes mellitus. J Nurs Sci (2012) 27(10):8–10. 10.3870/hlxzz.2012.19.008

[86] Zhu X Gong Y Yao S The effect of biofeedback assisted relaxation training on glucose metabolism in patients with type II diabetes: a follow-up study. Chin J Clin Psychol (2001) 9(1):4–9. 10.3969/j.issn.1005-3611.2001.01.002

[87] Zhu XZ Gong YX Yao SQ The effect of biofeedback assisted relaxation training on cytokines in patients with type II diabetes. Chin J Clin Psychol (2001) 9:170–2. 10.3969/j.issn.1005-3611.2001.03.004

