# Supplementary material for: Psychological Interventions for the Management of Glycemic and Psychological Outcomes of Type 2 Diabetes Mellitus in China: A Systematic Review and Meta-Analyses of Randomized Controlled Trials
Source: Front Public Health. 2015 Nov 16;3:252. doi: 10.3389/fpubh.2015.00252 (PMC4644788; doi:10.3389/fpubh.2015.00252)
Supplement: Supplementary file 1 [file Data_Sheet_1.DOCX]

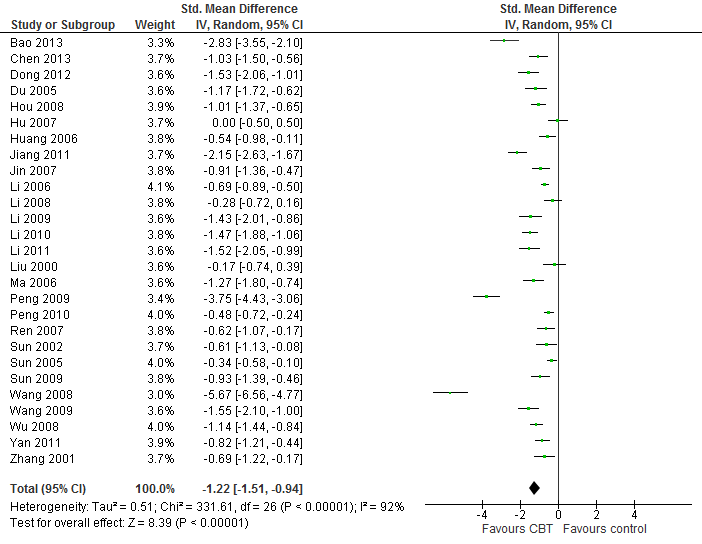


**Supplementary Figure 1. Meta-analysis illustrating the standardized effects of cognitive behavioral therapy (CBT) vs control for depression.**

**
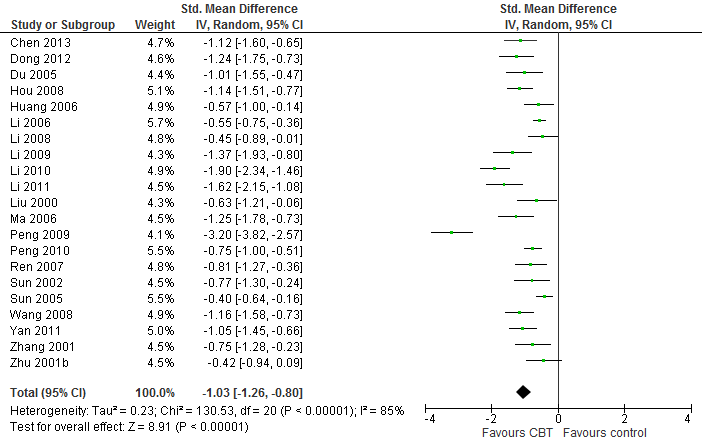
**

**Supplementary Figure 2. Meta-analysis illustrating the standardized effects of cognitive behavioral therapy (CBT) vs control for anxiety.**


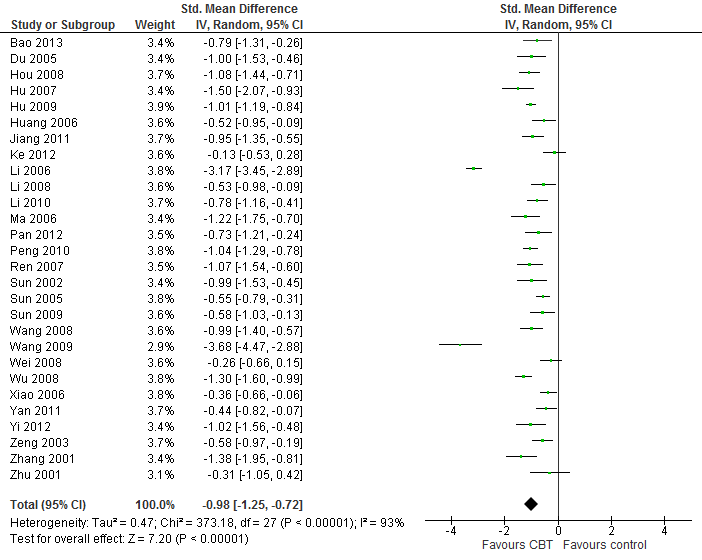


**Supplementary Figure 3. Meta-analysis illustrating the standardized effects of cognitive behavioral therapy (CBT) vs control for blood glucose concentration.**


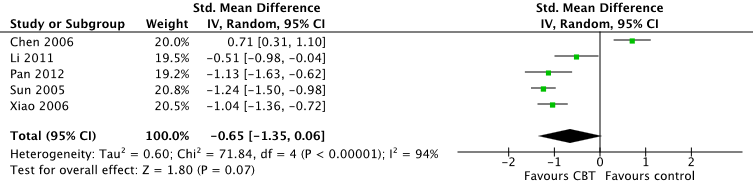


**Supplementary Figure 4. Meta-analysis illustrating the standardized effects of cognitive behavioral therapy (CBT) vs control for overall quality of life (QOL).**


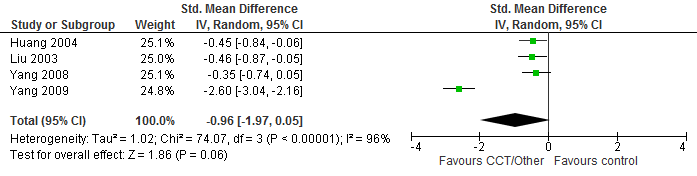


**Supplementary Figure 5. Meta-analysis illustrating the standardized effects of client-centered therapy (CCT) vs control for glycated hemoglobin.**

**
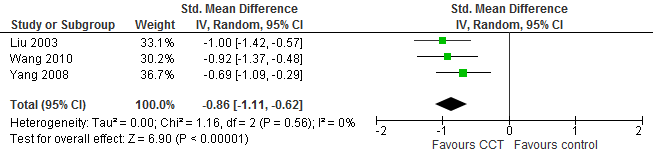
**

**Supplementary Figure 6. Meta-analysis illustrating the standardized effects of client-centered therapy (CCT) vs control for depression.**

**
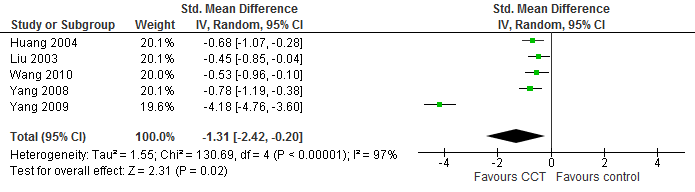
**

**Supplementary Figure 7. Meta-analysis illustrating the standardized effects of client-centered therapy (CCT) vs control for blood glucose concentration.**

**Publication Bias and Small Study Effects**

**CBT - Glycated Hemoglobin (HbA1c)**

**Egger's test for small-study effects:**

Number of studies = 20 Root MSE = 4.856

| Std_Eff | Coef. | Std. Err. | t | P>\|t\| | [95% Conf. Interval] |
| --- | --- | --- | --- | --- | --- |
| slope | -1.299146 | 0.5574103 | -2.33 | 0.032 | -2.470222 to -0.1280707 |
| bias | 1.561502 | 2.989731 | 0.52 | 0.608 | -4.719691 to 7.842695 |

Test of H0: no small-study effects P = 0.608

# CBT – Depression

**Egger's test for small-study effects:**

Number of studies = 27 Root MSE = 2.94

| Std_Eff | Coef. | Std. Err. | t | P>\|t\| | [95% Conf. Interval] |
| --- | --- | --- | --- | --- | --- |
| slope | 0.1944498 | 0.3246683 | 0.60 | 0.555 | -0.4742171 to 0.8631167 |
| bias | -5.784986 | 1.581809 | -3.66 | 0.001 | -9.042782 to -2.52719 |

Test of H0: no small-study effects P = 0.001

# CBT – Anxiety

**Egger's test for small-study effects:**

Number of studies = 21 Root MSE = 2.158

| Std_Eff | Coef. | Std. Err. | t | P>\|t\| | [95% Conf. Interval] |
| --- | --- | --- | --- | --- | --- |
| slope | -0.1171286 | 0.265258 | -0.44 | 0.664 | -0.6723201 to 0.4380628 |
| bias | -4.021107 | 1.338001 | -3.01 | 0.007 | -6.821576 to -1.220638 |

Test of H0: no small-study effects P = 0.007

# CBT – Blood Glucose Concentration

**Egger's test for small-study effects:**

Number of studies = 28 Root MSE = 3.787

| Std_Eff | Coef. | Std. Err. | t | P>\|t\| | [95% Conf. Interval] |
| --- | --- | --- | --- | --- | --- |
| slope | -1.043935 | 0.3880401 | -2.69 | 0.012 | -1.841562 to -0.2463066 |
| bias | 0.3326729 | 2.057039 | 0.16 | 0.873 | -3.895631 to 4.560976 |

Test of H0: no small-study effects P = 0.873
